# Comparison of biological activities of *Tityus pachyurus* venom from two Colombian regions

**DOI:** 10.1590/1678-9199-JVATITD-2021-0005

**Published:** 2021-12-06

**Authors:** Jennifer Alexandra Solano-Godoy, Julio César González-Gómez, Kristian A. Torres-Bonilla, Rafael Stuani Floriano, Ananda T. Santa Fé Miguel, Walter Murillo-Arango

**Affiliations:** 1 Natural Products Research Group (GIPRONUT), School of Sciences, University of Tolima, Altos de Santa Helena, Ibagué, Tolima, Colombia.; 2 Research Group BEA - Biology and Ecology of Arthropods, Corporación Huiltur, Neiva, Huila, Colombia.; 3 School of Sciences, University of Tolima, Altos de Santa Helena, Ibagué, Tolima, Colombia.; 4 Research Group on Bio-ecology of Vertebrates (BIVET), Fundación Merenberg, La Plata, Huila, Colombia.; 5 Department of Pharmacology, School of Medical Sciences, State University of Campinas (Unicamp), Campinas, SP, Brazil.; 6 Laboratory of Toxinology and Cardiovascular Research (LATEC), Graduate Program in Health Sciences, University of Western São Paulo (Unoeste), Presidente Prudente, SP, Brazil.

**Keywords:** Scorpion, Venom, Insecticides, Antibacterial agents, Chromatography, HPLC

## Abstract

**Background:**

In the present study, we have tested whether specimens of the medically relevant scorpion *Tityus pachyurus*, collected from two climatically and ecologically different regions, differ in the biological activities of the venom.

**Methods:**

Scorpions were collected in Tolima and Huila, Colombia. Chemical profiles of the crude venom were obtained from 80 scorpions for each region, using SDS-PAGE and RP-HPLC. Assays for phospholipase A_2_, direct and indirect hemolytic, proteolytic, neuromuscular, antibacterial, and insecticidal activities were carried out.

**Results:**

The electrophoretic profiles of venom from the two regions showed similar bands of 6-14 kDa, 36-45 kDa, 65 kDa and 97 kDa. However, bands between 36 kDa and 65 kDa were observed with more intensity in venoms from Tolima, and a 95 kDa band occurred only in venoms from Huila. The chromatographic profile of the venoms showed differences in the intensity of some peaks, which could be associated with changes in the abundance of some components between both populations. Phospholipase A_2_ and hemolytic activities were not observable, whereas both venoms showed proteolytic activity towards casein. Insecticidal activity of the venoms from both regions showed significant variation in potency, the bactericidal activity was variable and low for both venoms. Moreover, no differences were observed in the neuromuscular activity assay.

**Conclusion:**

Our results reveal some variation in the activity of the venom between both populations, which could be explained by the ecological adaptations like differences in feeding, altitude and/or diverse predator exposure. However more in-depth studies are necessary to determine the drivers behind the differences in venom composition and activities.

## Background

Scorpion venoms represent a complex mixture of neurotoxins, enzymes, proteins and peptides secreted by a specific gland inside the telson [1]. The neurotoxins mostly affect ion channels, mainly potassium (K^+^) and sodium (Na^+^) channels of neurons, and have been shown to play an important role in the ecology of scorpions, as well as in pharmacology and agricultural industry [1]. Scorpions have evolved specific toxins that specially affect ion channels of vertebrates or arthropods. Scorpions can therefore use their venoms to capture different types of arthropod preys, and also defend themselves from vertebrate predators [2]. In addition, ion channel toxins selective for arthropods can serve as templates for the development of new pesticides as sustainable solutions for the agricultural industry [3-6]. Furthermore, some peptides from scorpion venoms have gained interest in the pharmaceutical industry for their antimicrobial activities, due to the presence of antimicrobial peptides (AMPs). These peptides have been extensively evaluated as therapeutic agents based on their potent activities, low resistance rates and unique mechanisms of action [7-9].

Venom components may be more abundant in some individuals of a species than others due to the individual variability in the composition of venom. The variation occurring between individual venom samples can be a result of seasonal variation, diet, habitat, predator exposure, age and/or gender [10-12].

Intraspecific variation of venoms has been previously studied in different species of venomous animals, such as bees [13], wasps [14], ants [15], Cone shell snails [16], spiders [17], snakes [11,18] and scorpions [10,19-21]. Abdel-Rahman et al. [10], reported intraspecific variation in the Egyptian scorpion *Scorpio maurus palmatus* (Scorpionidae), and attributed it to environmental conditions or a reflection of genetic diversity among populations. Similarly, Estrada-Gómez et al. [20], reported intraspecific differences in the venom composition of *Centruroides edwardsii* from two regions of Colombia, which were attributed to an innate individual variation in the synthesis and expression of the venom. It therefore appears that local environmental conditions and geographical separation could play an important role in intraspecific variation.

The intraspecific variation in venom composition may have an effect on the biological activities, the mechanisms of action of the venom and on the symptomatology presented by the patients and their responses to antivenoms. Differences in bioactivity of the venom may be a heuristic approach for the effective bioprospecting of its metabolites [18,22]. 

*T. pachyurus* is a species of medical importance in Colombia, and its venom is considered one of the most toxic, and responsible for the highest percentage of scorpion sting patient admissions to hospitals in the country [11,23-25]. Envenomation by this species can present symptoms such as tachycardia, diaphoresis, tachypnea, cyanosis, hypertension, and bradycardia. In cases of severe scorpionism, cardiovascular compromise and cardiac arrest, and pulseless ventricular tachycardia are also present [26].

In this work, we compared the venom of *Tityus pachyurus* collected in two different departments in Colombia (Huila and Tolima), finding some differences in toxicity, enzymatic and biological activities of the venoms. This study represents a further advance towards the comprehension of the variability of venom composition of the medically important scorpion *T. pachyurus*.

## Methods

### Animal collection and housing

**Scorpions**

Eighty (80) adult scorpions per region were manually collected between the following hours: 20:00 and 00:00. We did not differentiate between males and females. All individuals collected were adults. Specimen collection was carried out under the permit granted by ANLA, resolution 00319 of March 29, 2017. 

**Figure 1.**
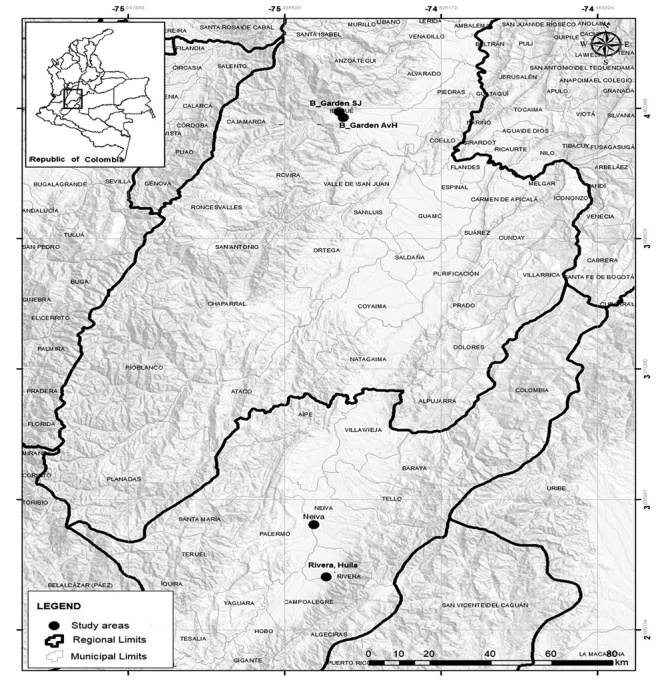
Location of sampling regions from Tolima and Huila departments in Colombia.

Scorpions were collected using ultraviolet lamps in different locations in diverse altitudes (Fig. 1):

- Ibagué, Tolima (1100 m ASL): Alexander Von Humboldt Botanical Garden (4° 25' 36.60'' N, 75° 12' 48.47'' W) and San Jorge Botanical Garden (4° 26' 52.50'' N, 75° 13' 34.10'' W).
- Rivera, Huila (920 m ASL): surroundings of Los Ángeles Termal (2° 45' 7.5" N, 75° 14' 15.4"W) and Granja Integral Modelo Experimental (2° 45' 43.43 N, 75° 15' 17.33 '' W).

After collecting the scorpions, they were transported to the Laboratory of the Natural Products Research Group (GIPRONUT, Universidad del Tolima). Each scorpion was kept individually in a plastic box with dry soil and a wet cotton to provide water. After experiments all the individuals were deposited in the Zoology Collection of the University of Tolima (CZUTARA-16D282DC7BD).

**Experimental animals**

Male Balb/c mice (25-30 g, 2-3 months old) obtained from the Multidisciplinary Center for Biological Investigation of the State University of Campinas (CEMIB/UNICAMP, Campinas, SP, Brazil) were housed in plastic cages (5-10/cage) with a wood-shaving substrate, at 23 ± 1 °C in ventilated stands (Alesco^®^) on a 12 h light/dark cycle with lights on at 6 a.m. Animals had free access to food and water. The animals were euthanized with isoflurane (saturated atmosphere) immediately prior to the experiments. The animal experiments were approved by an institutional Committee for Ethics in Animal Use of the State University of Campinas (CEUA/UNICAMP, protocol no. 5093-1/2018) and were done according to the general ethical guidelines for animal use established by the Brazilian Society of Laboratory Animal Science (SBCAL) and Brazilian legislation (Federal Law No. 11,794, of October 8, 2008), in conjunction with the guidelines for animal experiments established by the Brazilian National Council for Animal Experimentation (CONCEA) and EU Directive 2010/63/EU for the Protection of Animals Used for Scientific Purposes.

Cockroaches *Blaptica dubia* were used as a biological model in their third instar and kept in 12 Oz jars with food and a wet cotton to ensure humidity and water availability 

### Venom extraction

 Venom from *T. pachyurus* adult specimens was extracted by electrical stimulation on the metasoma through a power source (18 V, 40 Hz) [12,27]. The collected venom was pooled per region, transferred to low-protein binding tubes, lyophilized and stored at -20°C until their use.

### Venom characterization

The protein content quantification was determined in triplicate using standard curves of bovine serum albumin (BSA) between 0 and 125 mg/mL solutions, with absorbance being measured at 595 nm, as essentially described elsewhere [28].

**Sodium dodecyl sulphate-polyacrylamide gel electrophoresis (SDS-PAGE)**

The samples were separated in gels at 12% (v/v) following the protocol suggested by Yábar et al. [29], loading 8.53 µg protein per sample. Gels were run at a constant voltage of 100 V for 60 minutes, and were then stained with Coomassie Brilliant Blue R-250. The molecular mass was estimated with two Sigma-Aldrich (St. Louis, MO, USA) standard markers: C1992-1VL and S8445-1VL. 

**Reverse-phase high performance liquid chromatography (RP-HPLC)**

Reverse-phase HPLC (RP-HPLC) of *T. pachyurus* venom from Tolima and Huila region was carried out using a Discovery C18 column (250 mm x 4.6 mm x 5 µm; 300 Å) from Sigma-Aldrich Chemical Co. (St. Louis, MO, USA) coupled to a Shimadzu chromatographic system (Shimadzu, Tokyo, Japan) that consisted of two pumps (LC10AD VP), a UV/Vis detector (SPD-10A), a fraction collector (FRC-10A) and a system controller (SCL-10A VP). The column was initially equilibrated with 0.1% (v/v) trifluoroacetic acid (TFA). The venom (1 mg) was dissolved in ultrapure water with 0.1% (v/v) TFA and clarified by centrifugation (112 x g for 5 min). Proteins were eluted with a linear gradient (0-100%) of 99.9% acetonitrile in 0.1% TFA and the elution profile was monitored at 280 and 215 nm [30-32].

### Enzymatic activities

**Phospholipase A_2_ and hemolytic activity**

The venom samples were tested at different concentrations (0.25-10 μg/µL) with a lipoprotein solution from egg yolk in the presence of CaCl_2_. The hydrolysis of the phospholipids was detected after 15 minutes of incubation at 37 °C. It was standardized with 0.01 mol x l^-1^ sodium hydroxide (NaOH), and the results was expressed in μmol of fatty acid/mg of venom/minute [33].

Direct and indirect hemolytic activity was evaluated using agarose gels with red blood cells, in the presence or absence of calcium chloride (CaCl_2_) with egg yolk solution. Minimum hemolytic dose (MHD) was defined as the amount of venom that induced a 20 mm diameter hemolytic halo. As a positive control *Crotalus durissus* venom was used at a concentration of 2 μg/µL, and 0.25 M phosphate buffer pH 7.0 was used as a negative control [34].

**Proteolytic activity**

Casein (C7078; Sigma-Aldrich Chemical Co., St. Louis, MO, USA) was used as substrate to measure the proteolytic activity of all venoms, using the methodology reported by Kunitz [35] with modifications as follows: 250 μL of venom at different concentrations (0.5-4 μg/µL) selected according to preliminary analyses (data not shown) were added with 750 µL of casein at a concentration of 1% (v/v), the mixture was incubated for 15 min at 37 °C**,** after which the reaction was stopped with 30% (v/v) trichloroacetic acid (TCA) [36-38]. 

Samples were then centrifuged at 2800 x g for 10 min at 25 °C. The resulting proteolytic products were evaluated spectrophotometrically at ƛ = 280 nm. Results are shown as units of caseinolytic activity using the following formula: U/mg = (ΔA280/mg _venom_) x (100) which correspond to the amount that induces a change in absorbance of 0.001/minute. Each test was performed in triplicate and all reagents were analytical grade.

### Biological activities

**Bactericidal activity**

The bactericidal activity was assayed in 96-well microplates, in which 130 μL of the bacterial culture was placed in Brain Heart Infusion (BHI) with an initial absorbance at 610 nm in a Multiskan^TM^ Go microplate spectrophotometer and 70 μL of the respective venom. After 24 hours at 37 °C, bacterial growth was determined by the turbidity developed in each well. The inhibitory effect was determined by the absence of turbidity [39-41].

Bacterial strains obtained from the pathogenic strains collection of the Natural Products Research Group (GIPRONUT) of the University of Tolima were tested separately: *Escherichia coli* (ATCC25922), *Pseudomonas aeruginosa* (ATCC27853) and *Staphylococcus aureus* (ATCC29213).

**Insecticidal activity**

Insecticidal activity for *T. pachyurus* venoms was determined in cockroaches *Blaptica dubia*, as essentially described elsewhere [42-44]*.* Cockroaches (12/batch) were grouped according to similar mass ranges between 90 mg to 250 mg. Three replicates were performed for a total of 36 individuals per dose, with a venom exposure of 48 h. The venom was diluted to be administrated with 0.25 M phosphate buffer at pH 7.0 and tested with two doses (30 and 100 μg) through injection in the third segment of the ventral abdomen of each individual. As a positive control, cockroaches were treated with doses of 60 μg Malathion 57% EC ADAMA^®^ (commercial insecticide). As a negative control, cockroaches were treated with 0.25 M phosphate buffer at pH 7.0. Individuals were considered dead when they did not respond to any stimulus applied to the appendages with a brush, and mortality was recorded 24 hours and 48 hours after treatment. The batches were observed at 1 hour, 10 hours, 24 hours and 48 hours after envenomation. The median lethal time (LT_50_) and the median lethal dose (LD_50_) was estimated by Probit analysis.

**Mouse phrenic nerve-diaphragm (PND) preparations**

The hemidiaphragms and their respective phrenic nerves were dissected from male Swiss mice euthanized with isoﬂurane (saturated atmosphere) and then the preparations were mounted under a resting tension of 1 g in 5 mL organ baths containing aerated (95% O_2_ and 5% CO_2_) Tyrode solution (composition, in mM: NaCl 137, KCl 2.7, CaCl_2_ 1.8, MgCl_2_ 0.49, NaH_2_PO_4_ 0.42, NaHCO_3_ 11.9 and glucose 11.1, pH 7.0) at 37 °C and allowed to stabilize for 10 min prior to use, as described elsewhere [45]. Supramaximal stimuli (0.1 Hz, 0.2 ms) were delivered to the nerve from a Grass S88 stimulator (Grass Instrument Co., Quincy, MA, USA) and the muscle twitches were recorded using a TRI201AD force displacement transducer coupled to a Quad Bridge Amp and LabChart 6.0 software (all from ADInstruments Pty Ltd., Bella Vista, Australia). After stabilization, the preparations were incubated with *T. pachyurus* venom (10, 30 and 60 µg/mL) for 120 min or until complete neuromuscular blockade. Changes in the twitch-tension responses of PND preparations were expressed as a percentage relative to baseline (time zero) values.

**Neutralizing effect of scorpion antivenom from Brazil**

 The neutralizing action of scorpion antivenom produced by the Instituto Butantan (São Paulo, SP, Brazil) on the *T. pachyurus* venom-induced neuromuscular blockade in mouse PND preparation was tested by incubating venom at an antivenom:venom ratio of 1:1 (v/w). This ratio was based on the manufacturers stated neutralizing capacity for the antivenom in which 1 mL of antivenom neutralizes 1 mg of *Tityus serrulatus* venom. *T. pachyurus* venom from Huila and Tolima regions was pre-incubated with antivenom at 37 °C for 30 min before assaying for residual neuromuscular activity in PND preparation [45].

### Statistical analyses

 The results were expressed as the mean ± SDM. Statistical comparisons were made using a two-way ANOVA followed by the Tukey *post-hoc* test, with *p <* 0.05 indicating significance. The original data met the criteria of normal curve fit and homogeneity of variances.

## Results

### Characterization of venom: SDS-PAGE electrophoresis and chromatographic profile

**Figure 2:**
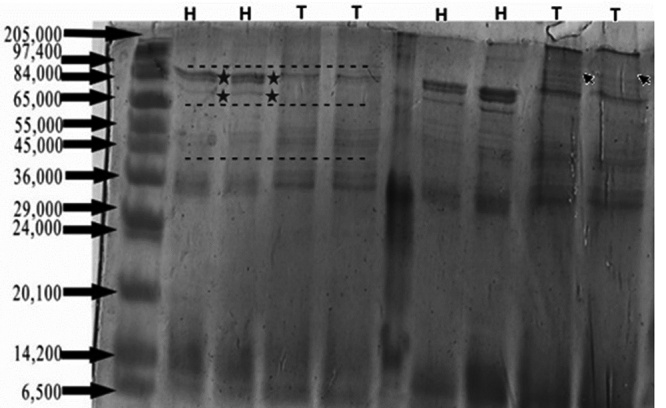
SDS-PAGE (12%) profile of venoms from Huila (H) and Tolima (T) regions. Stars indicate protein bands present in *T. pachyurus* venom from the Huila region and absent in *T. pachyurus* venom from Tolima. Arrows indicate protein bands present in *T. pachyurus* venom from Tolima and absent in venom from the Huila region.

**Figure 3.**
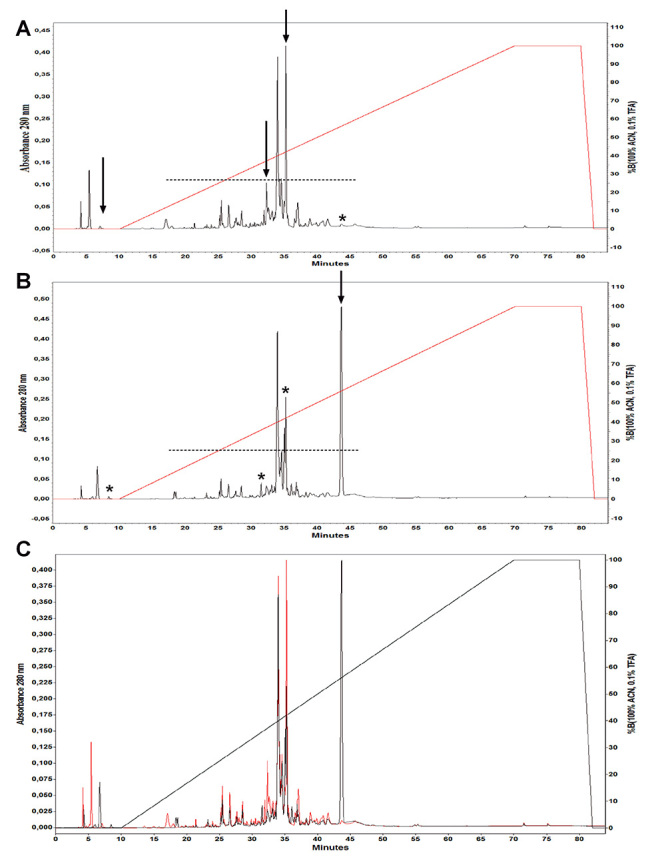
Separation by high performance liquid chromatography (RP-HPLC) of 1.0 mg of soluble venom of *Tityus pachyurus* from **(A)** Tolima and **(B)** Huila regions. The profile was performed on an inverted Discovery C18 analytical column equilibrated with solution A (water in 0.1% TFA) (v/v), the proteins were eluted with a linear gradient (0-100%) of 99.9% acetonitrile in 0.1% (v/v) TFA and the elution profile was monitored at 280 nm. Arrows indicate the presence of peaks with higher intensity; asterisks indicate the absence of peaks, or peaks with less intensity; and dotted lines indicate a conserved region in both venoms. **(C)** Overlap of the chromatographic profiles of *T. pachyurus* venom from Tolima (red line) and Huila (black line).

The electrophoretic profiles showed differences between scorpion venom from Huila and Tolima (Fig. 2). Bands close to 65 kDa were found only for Huila and bands close to 84 kDa and 97 kDa were found in less intensity for Tolima compared with Huila, bands close 55-36 kDa were found in less intensity for Huila compared with Tolima (Table 1). The RP-HPLC venom profile showed approximately 50 to 55 peaks and the major components were in a well conserved region in both venoms eluting in the range of 25.43 to 43.63 min elution time between 17% to 50 % of ACN (Fig. 3). 

**Table 1.** SDS-PAGE (12%) profile of scorpion venoms from Huila (H) and Tolima (T) regions. One plus (+) symbol indicates the band presence, two symbols (++) indicate more abundance of a band and a negative one (-) indicates the band absence.

Protein band (kDa)	Venom sample origin	
	Huila	Tolima
97.4	(++)	(+)
84	(++)	(+)
65	(+)	(-)
55	(+)	(++)
45	(+)	(++)
36	(+)	(++)
14.2	(+)	(+)
6.5	(+)	(+)

### Enzymatic activities

**Figure 4.**
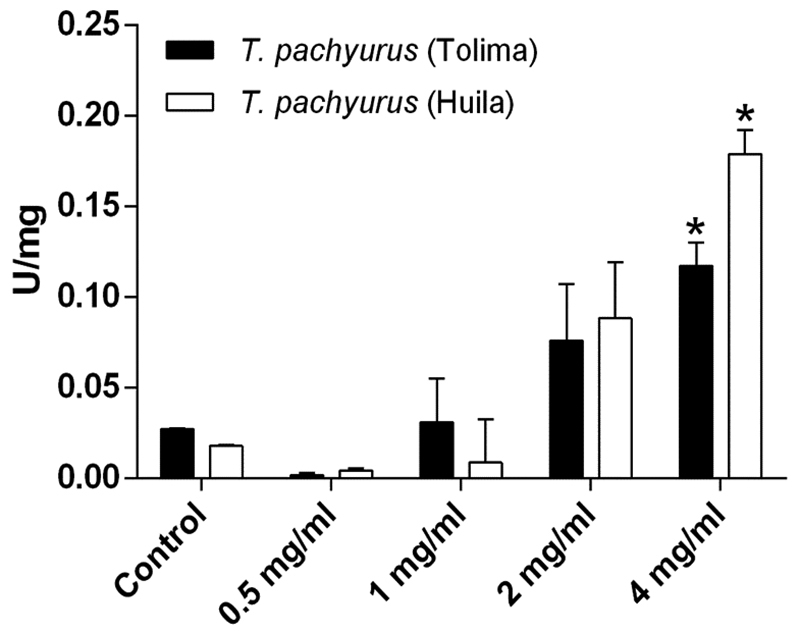
Proteolytic activity towards casein of venom from *Tityus pachyurus* from Tolima and Huila regions. *p < 0.05 two-way ANOVA compared to each other.

*T. pachyurus* venom did not show phospholipase A_2_ activity on egg yolk substrate nor indirect or direct hemolytic activity for either of the two regions. The venom from both regions showed proteolytic activity on casein substrate at doses higher than 1 mg/mL. The caseinolytic activity differed significantly (*p* ˂ 0.01) between Huila and Tolima at 4 mg/mL (Fig. 4).

### Biological activities

**Bactericidal activity**

The inhibition percentages were variable and less than 50% for the two venoms and a dose response effect was not evident. The Tolima venom showed a percentage of inhibition of bacterial growth of 36% against the gram-positive bacterium *S. aureus* while the venom from Huila showed inhibition of bacterial growth of 26% against the gram-negative bacterium *E. coli.* The two remaining strains did not show any type of inhibition. The data are shown in Table 2.

**Table 2.** Percentage of bactericidal inhibition caused by *T. pachyurus* venom from Tolima and Huila regions.

Bacterial strain	Sample	31 ppm	62 ppm	125 ppm	250 ppm	500 ppm
*Staphylococcus aureus*	**Tolima**	36.5 ± 15.48	43.4 ± 9.32	48.6 ± 5.89	21.2 ± 25.7	46.3 ± 5.46
	**Huila**	0	0.03 ± 2.53	0	0	0
*Pseudomona aeuroginosa*	**Tolima**	0.05 ± 0.05	0.05 ± 0.06	0.06 ± 0.03	0.06 ± 0.02	0.05 ± 0.08
	**Huila**	5.07 ± 2.69	12.71 ± 7.09	0	0	0
*Escherichia coli*	**Tolima**	0.32 ± 0.06	0.27 ± 0.06	0.19 ± 0.03	0.15 ± 0.06	0.15 ± 0.04
	**Huila**	26.17± 4.72	25.73 ± 1.38	22.55 ± 4.03	19.42 ± 8.571	21.32 ± 26.27

Data are reported as the mean ± SDM.

**Insecticidal activity**

We observed a difference in the toxicity between the venoms from Huila and Tolima. Venom from Huila presented insecticidal activity in *Blaptica dubia*. The cockroaches exposed to the treatment with the Tolima venom showed fewer envenomation symptoms, lower toxicity, and did not produce a significant mortality in the individuals exposed. It was not possible to calculate the LD_50_ for the Tolima venom, since none of the individuals died with the injected doses. On the other hand, the individuals exposed to venom from Huila showed a faster envenomation (LT_50_ of 52 min) and higher mortality (LD_50_ of 843.8 mg/kg).

The first symptoms of envenomation were evident one hour after treatment in the batches injected with Huila venom: uncoordinated displacement and rapid movements. After ten hours the cockroaches that remained alive responded to the stimulation of their appendices, but they did not have the capacity to displace; finally, after 24 hours there were signs of mortality.

**Neuromuscular activity and neutralization**

**Figure 5.**
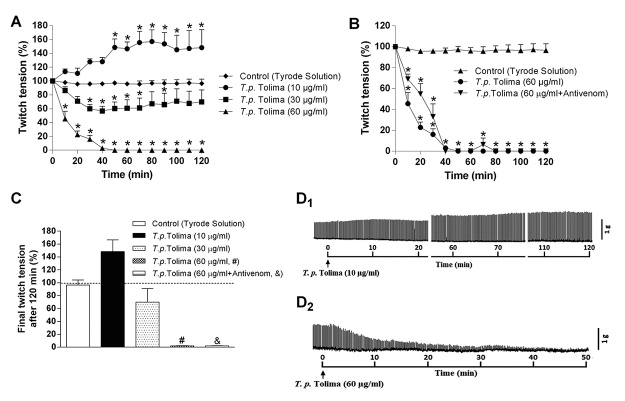
Neuromuscular activity of *T. pachyurus* venom from Tolima in PND preparations. **(A)** Neuromuscular effects induced by the venom at different concentrations. It was observed complete blockade with the highest concentration and facilitation with the lowest concentration after 120 min of incubation. **(B)** Failure of the scorpion antivenom from Brazil to inhibit the complete blockade induced by the venom. **(C)** Final twitch tension after 120 min of incubation with all the concentrations and antivenom. Representative recordings showing the facilitation induced by **(D**_1_ **)** the lowest concentration of venom and **(D**_2_ **)** the complete blockade produced by the highest concentration of venom. The points in **A** and **B** and columns in **C** are the mean ± SEM (n = 4). *p < 0.05 two-way ANOVA compared to control preparations.

*T. pachyurus* venom (10 µg/mL) from Tolima caused progressive neuromuscular facilitation which reached 48 ± 12% of increasing in twitch amplitude after 120 min incubation (*p* < 0.05 compared to control preparations, n = 4); venom produced concentration-dependent neuromuscular blockade with the intermediary concentration (30 µg/mL) decreasing 30 ± 17% of twitch responses after 120 min incubation and the highest concentration (60 µg/mL) causing complete blockade in ~40 min incubation (times for 50% and 90% blockade: ~10 and ~38 min, respectively; *p* < 0.05 compared to control preparations, n = 4); there was no evidence for neuromuscular facilitation with the 30 and 60 µg/mL venom concentrations (Fig. 5A, Table 3). The neuromuscular blockade induced by *T. pachyurus* venom from Tolima (60 µg/mL) was not neutralized by scorpion antivenom (Fig. 5B). 

**Table 3.** Comparison of the potency of *Tityus pachyurus* and other *Tityus* venoms determined as the time required to induce neuromuscular blockade in mice phrenic nerve-diaphragm (PND) preparations. The t_50_ and t_90_ values were estimated from graphs provided in the cited publications.

Scorpion specie	Venom concentration (µg/mL)	T_50_ (min)	T_90_ (min)	Facilitation (%)	Reference
*Tityus pachyurus* (Huila-Colombia)	10	NA	NA	~60	This work
	30	NA	NA	NA	
	60	~18	~90	NA	
*Tityus pachyurus* (Tolima-Colombia)	10	NA	NA	~40	This work
	30	NA	NA	NA	
	60	⁓10	⁓38	NA	
*Tityus bahiensis* (Brazil)	1	NA	NA	~75	Collaço et al. [71]
	3	NA	NA	~30	
	10	NA	NA	~50	
	30	~20	~40	~75	
*Tityus serrulatus* (Brazil)	0,5	NA	NA	~250	Borja-Oliveira et al. [68]
*Tityus cambridgei* (Brazil)	10	NA	NA	~75	Borja-Oliveira et al. [68]

NA: not applicable (the venom concentration did not induce 50 or 90% neuromuscular blockade during the frame of the experiment).

In summary, Figure 5C shows the endpoints recorded after 120 min incubation with Tyrode solution (control), venom (10-60 µg/mL) and venom pre-incubated with antivenom. Figure 5D_1_ shows the representative recording of the *T. pachyurus* venom from Tolima (10 µg/mL)-induced neuromuscular facilitation whereas the Figure 5D_2_ is a representative recording of the *T. pachyurus* venom from Tolima (60 µg/mL)-induced complete neuromuscular blockade in PND preparation.

**Figure 6.**
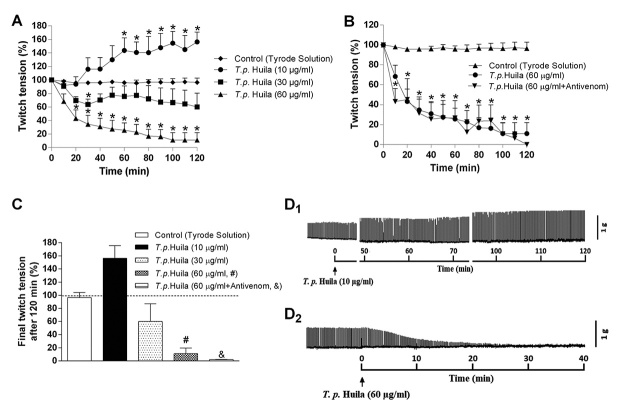
Neuromuscular activity of *T. pachyurus* venom from Huila in PND preparations. **(A)** Neuromuscular effects induced by the venom at different concentrations. It was observed complete blockade with the highest concentration and facilitation with the lowest concentration after 120 min of incubation. **(B)** Failure of the scorpion antivenom from Brazil to inhibit the complete blockade induced by the venom. **(C)** Final twitch tension after 120 min of incubation with all the concentrations and antivenom. Representative recordings showing the facilitation induced by **(D**_1_ **)** the lowest concentration of venom and **(D**_2_ **)** the complete blockade produced by the highest concentration of venom. The points in **A** and **B** and columns in **C** are the mean ± SEM (n = 4). *p < 0.05 two-way ANOVA compared to control preparations.

*T. pachyurus* venom (10 µg/mL) from Huila also produced facilitatory responses reaching 56 ± 14% of increase in twitch amplitude after 120 min incubation (*p* < 0.05 compared to control preparations, n = 4). In PND preparations exposed to 30 and 60 µg of venom/mL, there was a concentration-dependent neuromuscular blockade with the intermediary concentration inducing partial blockade of the twitch responses (40 ± 20% of blockade after 120 min incubation, n = 4), whereas the highest concentration caused complete blockade after approximately 100 min incubation (times for 50% and 90% blockade: approximately 18 and 90 min, respectively; *p* < 0.05 compared to control preparations, n = 4); *T. pachyurus* venom (30 and 60 µg/mL) from Huila also did not produce neuromuscular facilitation prior to blockade (Fig. 6A, Table 2).

The neuromuscular blockade induced by *T. pachyurus* venom from Tolima (60 µg/mL) was not neutralized by scorpion antivenom (Fig. 6B). In summary, Figure 6C shows the endpoints recorded after 120 min incubation with Tyrode solution (control), venom (10-60 µg/mL) and venom pre-incubated with antivenom. Figure 6D_1_ shows the representative recording of the *T. pachyurus* venom from Huila (10 µg/mL)-induced neuromuscular facilitation, whereas Figure 6D_2_ is a representative recording of the *T. pachyurus* venom from Huila (60 µg/mL)-induced complete neuromuscular blockade in PND preparation.

**Figure 7.**
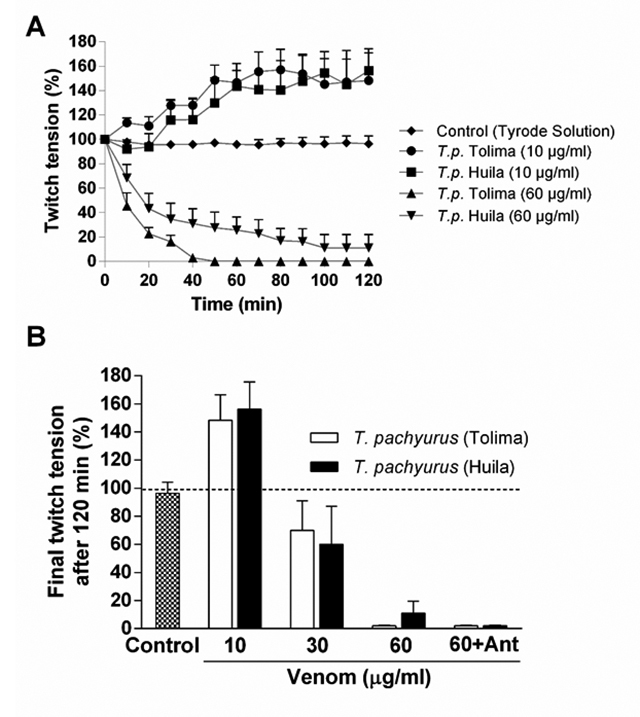
(A) Comparative neuromuscular activity between *T. pachyurus* venom from Tolima and Huila regions. (B) Comparative final twitch tension responses after 120 min incubation with venom from both regions and neutralization with scorpionic antivenom from Brazil. Note that there is not any significant difference in the neuromuscular activity caused by the venom from both regions as well as with the neutralization with antivenom. The points in A and columns in B are the mean ± SEM (n = 4).

*T. pachyurus* venoms from Huila and Tolima produced similar effects in PND preparations characterized by a progressive neuromuscular facilitation (~48-56% of increase in twitch amplitude) seen with the lowest concentration (10 µg/mL), whereas the intermediary concentration (30 µg/mL) caused partial neuromuscular blockade (~30% of reduction in twitch amplitude) after 120 min incubation. The highest concentration (60 µg/mL) produced complete neuromuscular blockade within ~40-90 min (Fig. 7). The complete neuromuscular blockade seen with by both of these venoms was not affected by pre-incubation with scorpion antivenom.

Histological analysis of the preparations treated with *Tityus pachyurus* venom from Huila and Tolima did not reveal any morphological alteration, with no morphological difference compared to those control preparations incubated with Tyrode solution (data not shown).

## Discussion

Crude venom SDS-PAGE shows a high concentration of protein content with molecular mass between 34 kDa and 97 kDa and other ones with higher concentration between 14 kDa and 7 kDa, these results coincide with bands reported by Barona et al. [23]. Tolima and Huila crude venoms showed variations in relative amounts of protein bands, these results are in concordance with those reported in other scorpions and arachnids where the components of the venom of a species may be more abundant in some individuals than in others due to individual variability in the composition of the venom [10,20,46,47] where aspects such as geographic distribution, diet, climate, age, and sex have been an integral part of determining the venom components [46,48,49]. The chromatographic profiles of the venoms from the Huila and Tolima regions showed a difference in the peaks and their intensity. Essentially, well defined peaks eluting at 8.37 min, 25.43 min, and 35.34 min presented in the venom from Tolima were visible with much less intensity in the venom from Huila (Fig. 3). The main peak in the Huila venom eluting at 43.40 min is missing in the Tolima venom. These findings could suggest a difference in the concentration of some of the components of the samples of these venoms from both regions.

The enzymatic profiles were similar to reported by Venancio et al. [50] and Almeida et al. [51], who also did not find phospholipase A_2_ enzymes in venoms from *Tityus* spp.*, T. serrulatus, T. bahiensis and T. stigmurus.* On the other hand, proteolytic enzymes in scorpion venoms are associated with the activation of coagulation cascades, fibrinolytic activity, homeostatic imbalance or pre-digestion of preys [51] and these enzymes probably process and activate toxins and trypsinogen which contributing to the pancreatitis that is often observed in the victims of attacks by *Tityus*, by increasing the concentration of enzymes in the pancreatic secretion [50,52].

Serine proteases, metalloproteases and hyaluronidases have been reported for the genus *Tityus*. In *T. discrepans*, serine and metalloproteases were found associated with fibrinolytic activity and anticoagulant effect. An anterease type metalloproteinase was reported for *T. pachyurus* named: TpachMP_A2 (UniProt V9ZAX6), which is capable of penetrating intact tissue and specifically cleaving soluble N-ethylmaleimide-binding protein (SNARE) receptors involved in pancreatic secretion, thereby disrupting normal vesicular traffic [53,54]. The caseinolytic activity can be attributed to protease-type enzymes in the venom of *T. pachyurus*.

Despite the low level of inhibition, the results obtained through the bactericidal assays suggest that the crude venom has a direct effect against the bacterial growth or integrity of the gram-negative bacterium *E. coli.* and gram-positive *S. aureus.* Antimicrobial peptides derived from scorpion venom have gained great interest due to their potent activity, low rates of resistance and a unique mode of action, which have increased the interest of the pharmacological industry [8,9,55-58]. Duenas-Cuellar et al. [59] reported a toxin from *Centruroides margaritatus* venom that inhibiting the proliferation of the gram-negative bacteria *Klebsiella pneumoniae*. For the genus *Tityus*, these types of peptides for *T. serrulatus, T. discrepans, T. costatus* and *T. obscurus* are registered in the UniProt Consortium 2002-2018 database. [60-62]. For this reason, it is important not to rule out exploring other forms of antimicrobial activity such as fungal activity and it is suggested to carry out bactericidal activity tests with fractions of the *T. pachyurus* venom.

The insecticidal activity showed marketable differences between venom from Tolima and Huila. Only the venom extracted from Huila individuals showed insecticidal activity on *B. dubia.* Scorpions are animals that survive in extreme and adverse physical conditions, with nocturnal habits and remain hidden during the day, feeding on small insects [63]. According to literature, their venoms are considered a rich source of different compounds, however the best-known toxins are those specific for Na^+^ or K^+^ channels (NaScTxs and KTxs, respectively). These peptides act on ion channels with high affinity and specificity, and may also discriminate between ion channels of insects and mammals and can cause death or rapid paralyze of the prey by interacting with ion channels and/or receptors in the neuromuscular system as observed with the venom of Huila scorpions. Anti-insect toxins have been previously reported from Colombia scorpions: Rincon-Cortés et al. [64] reported anti-insect toxins in the venom of *Tityus macrochirus* from Colombia and Guerrero-Vargas et al. [32] reported the first anti-insect excitatory β class NaScTx in the venom of *T. pachyurus* from Colombia, the differences between venom from Huila and Tolima regions should be deeply analyzed and could be associated with the variation in the chemical composition evidenced through the chromatographic analysis and SDS-PAGE carried out, however it is necessary to go deeper from a proteomic, transcriptomic and metabolomics approaches, and analyze the ecology in more details the ecology from both regions. 

*T. pachyurus* venom from Huila and Tolima regions produced the same effect in PND preparations without any significant difference, both venoms produced ⁓48-56% facilitation with the lowest concentration tested (10 µg/mL) and complete blockade with the highest concentration (60 µg/mL) after ⁓40-90 min. The neuromuscular action of *T. pachyurus* venom demonstrated in this present study comprises the first report with Colombian scorpion species. The action of *Tityus* venoms on the somatic neurotransmission has been poorly demonstrated in the last decades, with only a few studies reporting their neurotoxic mechanisms in vertebrate nerve-muscle preparations [65-71]. The neuromuscular facilitation produced by *T. pachyurus* venom at low concentrations and the rapid blockade of the twitch responses seen at high concentrations of this venom in PND preparations also were reported for the Brazilian species *T. serrulatus* [67] and *T. bahiensis* [70]. The neuromuscular action of scorpion venoms has been mostly associated with the presence of neurotoxins with high affinity for voltage-gated Na^+^ channels [70-71].

## Conclusions

The results obtained in the biological and enzymatic activities suggest a difference between venoms from Huila and Tolima regions, which could be an indication of variation in the venom composition of *T. pachyurus*. Currently, there is no geographical barrier between the region of Tolima and Huila and, probably, intraspecific variation of *T. pachyurus* venom is a consequence of a combination of local environmental conditions and differences in the type of diet or/and predators. This study represents an advance towards the understanding of the variability in the venom of *T. pachyurus.*
